# GPS-SUMO: a tool for the prediction of sumoylation sites and SUMO-interaction motifs

**DOI:** 10.1093/nar/gku383

**Published:** 2014-05-31

**Authors:** Qi Zhao, Yubin Xie, Yueyuan Zheng, Shuai Jiang, Wenzhong Liu, Weiping Mu, Zexian Liu, Yong Zhao, Yu Xue, Jian Ren

**Affiliations:** 1 State Key Laboratory of Biocontrol, School of Life Sciences, School of Advanced Computing, Sun Yat-sen University, Guangzhou 510275, China; 2 Department of Biomedical Engineering, College of Life Science and Technology, Huazhong University of Science and Technology, Wuhan 430074, China

## Abstract

Small ubiquitin-like modifiers (SUMOs) regulate a variety of cellular processes through two distinct mechanisms, including covalent sumoylation and non-covalent SUMO interaction. The complexity of SUMO regulations has greatly hampered the large-scale identification of SUMO substrates or interaction partners on a proteome-wide level. In this work, we developed a new tool called GPS-SUMO for the prediction of both sumoylation sites and SUMO-interaction motifs (SIMs) in proteins. To obtain an accurate performance, a new generation group-based prediction system (GPS) algorithm integrated with Particle Swarm Optimization approach was applied. By critical evaluation and comparison, GPS-SUMO was demonstrated to be substantially superior against other existing tools and methods. With the help of GPS-SUMO, it is now possible to further investigate the relationship between sumoylation and SUMO interaction processes. A web service of GPS-SUMO was implemented in PHP + JavaScript and freely available at http://sumosp.biocuckoo.org.

## INTRODUCTION

By covalently modifying specific lysine residues in protein substrates, or by non-covalently interacting with proteins, small ubiquitin-like modifiers (SUMOs) play an essential role in the regulation of a variety of biological processes, including gene expression, DNA repair, chromosome assembly, and cellular signaling (1–4). Along with the accumulating research on its biological functions, there are abundant evidences that the aberrance of SUMO regulation is highly associated with various diseases, such as neurodegenerative diseases (5,6), congenital heart defects (7), diabetes (8) and cancers (9). Therefore, the identification of SUMO modification sites and SUMO-interaction motifs (SIMs) in proteins is fundamental for understanding the biological functions and regulatory mechanisms of SUMOs, and provides potential targets for further diagnostic and therapeutic consideration.

The process of proteins being covalently modified by SUMOs is called as sumoylation, which is one of the most important and ubiquitous post-translational modifications (PTMs) of proteins (10,11). Previously, experimental studies suggested that most of sumoylation sites follow a canonical consensus motif of ψ–K–X–E (ψ, a hydrophobic amino acid, such as A, I, L, M, P, F, V or W; X, any amino acid residue) (12,13). However, our collective experimental data shows that approximately 40% (400 out of 983 sites) of known sumoylation sites do not conform to the above motif (Supplementary Table S1 and 3). In this regard, the current understanding of sumoylation recognition is still inadequate.

Recently, it was reported SUMOs can non-covalently interact with other proteins through targeting specific SIMs, which were also called as SUMO-binding motifs (SBMs) (14–16). For example, the SUMO interaction of Daxx modulates its sumoylation and is critical for targeting Daxx to PML oncogenic domains (PODs) for the transcriptional repression (17). Also, the non-covalent interaction of SUMO-2 and CoREST1, but not the sumoylation, is essential for organizing the transcriptional corepressor complex of LSD1/CoREST1/HDAC (18). Previously, a series of SIMs were experimentally identified (14,15,18–22). In early 2000, Minty *et al.* reported that the X{4,13}LSX[ST] motif appeared to be crucial for the SUMO interaction (19). However, four years later, Song *et al.* refuted the dominant role of serine residue in the SIMs (20), and discovered a new hydrophobic core motif of [VI]X[VI][VI] as a *bona fide* SIM (21). Compared to diverse ubiquitin-binding domains (UBDs) that commonly interact the β-sheet around Ile44 of ubiquitin (23,24), Song *et al.* demonstrated a novel mechanism for SUMO interaction, by forming an extended structure to bind between the α-helix and a β-strand of SUMO-1 (21). Thus, an in-depth study of SIMs can be not only helpful for distinguishing the distinct regulations between SUMOs and ubiquitin, but also provide implications for identifying non-covalent interactions of other ubiquitin-like (UBL) proteins. Later studies (14,15) confirmed the importance of consecutive hydrophobic residues, and raised a number of other SIMs, such as [VILMFWA][VILMFWA]XSX[ST][DE][DE][DE] and KX{3,5}[VI][IL][IL]XXX[DEQN][DE][DE], can also facilitate the SUMO interaction. In 2009, Ouyang *et al.* showed that an unusual type of SIM as [IVL][DE][IVL][DE][IVL] mediates the SUMO-2 specific interaction in several proteins (18). Subsequently, three additional types of SIMs were described as [PILVM][ILVM]X[ILVM][DSE>]{3}, [PILVM][ILVM]DLT and [DSE]{3}[ILVM]X[ILVMF]{2} by Vogt *et al.* (22). Although nearly ten types of SIMs were experimentally identified, each one can only recall a small proportion of known SIMs, and no one can present a major profile for SIMs.

Because of the complicated features, systematic analysis of sumoylation and SUMO interaction is still a great challenge. In contrast with labor-intensive and time-consuming experimental identifications, *in silico* prediction of sumoylation sites and SIMs in proteins can greatly narrow down the number of candidates, and generate helpful information for further verification. In the past decade, our group has made great efforts in developing a series of tools for predicting protein sumoylation sites (25,26). In early 2006, we released an online service of SUMOsp based on the first-generation Group-based Prediction System (GPS) algorithm (25). Subsequently, a matrix mutation (MaM) method was integrated into SUMOsp to upgrade the performance and a new version of SUMOsp 2.0 was introduced (26). In addition, other researchers have also constructed several reliable tools for the prediction of sumoylation sites, including SUMOplot (http://www.abgent.com/sumoplot), seeSUMO (27), SUMOpre (28) and SUMOhydro (29). However, these tools only focused on the sumoylation prediction, while a predictor for SIMs is still need to be developed.

In this work, by improving the prediction algorithm and adding the novel SIM prediction feature, we developed an updated version of SUMOsp and renamed it as GPS-SUMO. From the scientific literature, we manually collected 983 sumoylation sites in 545 proteins and 137 known SIMs in 80 proteins as the non-redundant data sets, respectively. Subsequently, the fourth-generation GPS algorithm integrated with the PSO (30,31) method was employed for training and predicting. For convenience, a user-friendly web interface was developed using PHP + JavaScript, and is freely available at http://sumosp.biocuckoo.org.

## IMPLEMENTATION

By searching the scientific literature (published before September 2013) in the PubMed with the keywords of ‘SUMO’, ‘sumoylation’ and ‘sumoylated’, we updated our previous training data set. The latest data set contained 1059 sumoylation sites in 594 proteins and 151 SIMs in 88 proteins. An online database of these experimentally verified sites was then developed and the intact annotations from UniProt and NCBI were integrated. To avoid overestimation of the prediction accuracy, the redundant sites should be removed, and the CD-HIT (32) with a threshold of 40% sequence identity was used to single out homologous proteins. If two proteins were found to be modified at the same position and to have more than 40% sequence identity, only one of the two proteins was preserved. In particular, 71 sumoylation sites were randomly picked from the latest collected data set to construct an additional testing data set. Due to the data limitations, an additional testing data set for SUMO interaction was not constructed. Finally, 912 sumoylation sites in 510 protein (Supplementary Table S1) and 137 SIMs in 80 proteins (Supplementary Table S2) were retained as the non-redundant training data sets.

In GPS-SUMO, the fourth-generation GPS algorithm was applied to predict the potential sumoylation sites and SIMs. As previously reported, consecutive hydrophobic residues are crucial for the non-covalent SUMO interaction (14,15). Based on the experimental observations, we summarized a hydrophobic motif of [IVL]{3,5} from the experimentally verified SIMs. Only 5 (∼3.6%) of 137 known SIMs did not follow this pattern (Supplementary Table S2). This motif was then integrated into the GPS algorithm to filter potentially false positive hits for the SIMs prediction. To enhance the prediction accuracy, the GPS algorithm adopted an additional procedure with four sequential training steps: *k*-means clustering, motif length selection (MLS), weight training (WT) and matrix mutation (MaM) (33). In the previous version (33), WT and MaM were implemented in a random mutation algorithm that required a long time for training and frequently resulted in a non-convergent result. Therefore, in the fourth-generation GPS algorithm, the Particle Swarm Optimization (30,31) algorithm was integrated to accelerate the training processes of WT and MaM steps and greatly improve the prediction performance. Before describing the PSO improvement, we re-illustrated the WT process as shown in Equation (1):

(1) \begin{equation*}
w_i = 1 + \Delta w_i
\end{equation*}

The *w_i_* was the scoring weight and Δ*w_i_* represented the numeric changes in the scoring weight after the training process. The WT process was aimed at finding a set of Δ*w_i_* with the optimal performance. Similarly, the MaM process can also be described as shown in Equation (2):

(2) \begin{equation*}
S(a,b) = Score(a,b) + \Delta S(a,b)
\end{equation*}

The *S*(*a,b*) was the optimal substitute score for an amino acid pair of *a* and *b*, while *S*(*a,b*) was the substitute score in the BLOSUM62 matrix. The *ΔS(a,b)* represented the numeric changes in the substitution score for an amino acid pair of *a* and *b*. Thus, the MaM approach sought a set of *ΔS(a,b)* that maximized the prediction performance. In the first step of PSO, a population array of particles with random current positions and velocities was initialized. For each particle, the leave-one-out (LOO) validation was used to evaluate the prediction performance in the current position. In this case, the randomly generated Δ*w_i_* and Δ*S*(*a,b*) were directly assigned to the current position. Next, the particles in the population communicated with each other through a neighborhood structure with a ring topology. Guided by the best position found in a specific neighborhood, a particle moved to a new position that much closer to the globally optimal one. By iteratively performing the above steps until a convergence criterion was met, an optimized solution for WT and MaM was achieved and a more accurate predictive model was obtained in GPS-SUMO. Also, the training efficiency of GPS-SUMO was considerably improved. More details on the fourth-generation GPS algorithm were provided in the Supplementary Methods.

To construct the online service of GPS-SUMO, we developed an easy-to-use web interface using PHP and JavaScript. For convenience, we tested the online service on a variety of internet browsers, including Internet Explorer, Mozilla Firefox and Google Chrome. To support the large-scale prediction, stand-alone packages were also developed in Java SE 6 and supported by Windows, Linux and Mac OS.

## RESULTS

For the preparation of training data sets, we took known sumoylation sites as the positive dataset, while all other non-sumoylated lysines in the same substrates were taken as the negative dataset. Similarly, the experimentally identified SIMs were regarded as positive data, while all other unidentified SIMs following the [IVL]{3,5} motif in the same proteins were taken as negative data. More details were shown in Supplementary Methods. Totally, the non-redundant training data set of sumoylation included 912 positive sites and 24491 negative sites (Supplementary Table S1), and ∼60% of all known sites are consensus sites (Supplementary Figure S1A). In SUMOsp 2.0, we only classified known sumoylation sites into two clusters, including the consensus group with sites following the ψ–K–X–E motif and the non-consensus group (26). In this work, we further used the *k*-means clustering approach to group non-consensus sites into two clusters, and demonstrated that such a procedure considerably improved the prediction performance. More clusters will result in fewer number of non-consensus sites in each cluster, and make the predictive model unstable. The sequence logos were illustrated by Weblogo (34) for each cluster (Supplementary Figure S1). For the two non-consensus groups, one cluster considerably enriches sites following the ψ–K–X–D motif (Supplementary Figure S1B), whereas the sequence profile is ambiguous for the other cluster (Supplementary Figure S1C). For SUMO interaction, the training data set contained 137 positive sites and 1699 negative sites.

**Figure 1.**
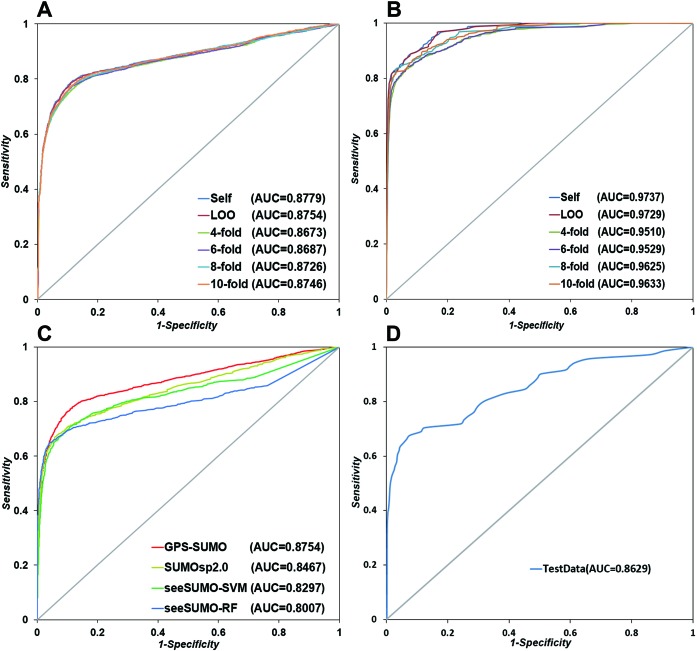
Performance evaluation of GPS-SUMO. (**A**) The performance evaluation for the prediction of sumoylation sites. The Self, LOO and *n*-fold validations were carried out. (**B**) The performance evaluation for the prediction of SIM. (**C**) A performance comparison among GPS-SUMO, SUMOsp 2.0 and seeSUMO. The algorithms of SVMs and RF in seeSUMO were separately validated and compared. The LOO validation was carried out in this comparison. (**D**) A further evaluation of the sumoylation prediction. An additional test data set was applied to perform this evaluation.

To evaluate the prediction performance of GPS-SUMO, we performed the self-consistency validation (Self), LOO validation and *n*-fold cross-validations (*n*-fold) of the training data set. For each validation, the sensitivity (23), specificity (*Sp*), accuracy (*Ac*), Mathew correlation coefﬁcient (*MCC*) and precision (*Pr*) were calculated. The receiver operating characteristic (ROC) curves were drawn. Also, the values of area under the curve (AUC) were calculated. For sumoylation site prediction, the AUC values of Self, LOO, 4-, 6-, 8- and 10-fold validations were 0.8779, 0.8754, 0.8673, 0.8687, 0.8726 and 0.8746, respectively (Figure 1A). Also in the SIM prediction, the AUC scores were calculated as 0.9737 for Self, 0.9729 for LOO, 0.9510 for 4-fold, 0.9529 for 6-fold, 0.9625 for 8-fold and 0.9633 for 10-fold validations (Figure 1B). The similar results of different validations demonstrated that GPS-SUMO is a stable and robust prediction tool. Based on the above evaluation, the three thresholds of high, medium and low stringency were chosen for GPS-SUMO. In order to balance the prediction performance, the medium stringency was selected as the default threshold.

In order to demonstrate the superiority of GPS-SUMO, we compared the prediction performance of GPS-SUMO with SUMOsp 2.0 and other existing tools or methods. Because SUMOsp 2.0 was demonstrated to be better than SUMOplot and SUMOpre (26), and SUMOhydro (29) did not support the batch prediction for multiple sequences, here we only compared GPS-SUMO with a recently released predictor of seeSUMO (27). To avoid any bias, the same training data set used in GPS-SUMO was adopted in SUMOsp 2.0 and seeSUMO. For comparison, the LOO validation was carried out, and the ROC curves were plotted in Figure 1C. In our results, the AUC values were calculated as 0.8754 for GPS-SUMO, 0.8467 for SUMOsp, 0.8297 for the seeSUMO with the Support Vector Machines (SVMs) algorithm and 0.8007 for the seeSUMO with the Random Forest (RF) algorithm, respectively (Figure 1C). Thus, the GPS-SUMO exhibited superior than other tools, and the PSO algorithm did increase the prediction accuracy. For SUMO interaction, a comparison was carried out between the GPS-SUMO and other experimentally verified motifs (Table 1). We fixed the *Sp* value of the GPS-SUMO to compare the *Sn* and *Ac* scores with these motifs. Obviously, the GPS-SUMO generate much better performance than the motifs (Table 1).

**Table 1.** Performance comparison of GPS-SUMO with known motifs for predicting SIMs

Motif^a^	Motif performance					GPS-SUMO performance				
	*Ac*	*Sn*	*Sp*	*MCC*	*Pr*	*Ac*	*Sn*	*Sp*	*MCC*	*Pr*
Motif 1	86.34%	17.88%	87.30%	0.0182	1.94%	87.34%	92.05%	87.30%	0.2028	5.25%
Motif 2	98.63%	1.99%	99.99%	0.1207	75.00%	99.26%	3.31%	99.99%	0.1528	71.43%
Motif 3	98.46%	19.87%	99.57%	0.273	39.47%	99.29%	64.24%	99.56%	0.5784	52.72%
Motif 4	97.74%	47.02%	98.45%	0.3644	38.82%	99.24%	72.19%	99.45%	0.5972	50.00%
Motif 5	98.59%	1.32%	99.96%	0.0641	33.33%	99.28%	10.60%	99.96%	0.2639	66.67%
Motif 6	98.62%	3.31%	99.96%	0.1332	55.56%	99.28%	10.60%	99.96%	0.2639	66.67%
Motif 7	98.62%	8.61%	99.89%	0.2076	52.00%	99.29%	20.53%	99.89%	0.3472	59.62%
Motif 8	98.70%	8.61%	99.97%	0.262	81.25%	99.28%	10.60%	99.96%	0.2639	66.67%
Motif 9	98.62%	6.62%	99.92%	0.1832	52.63%	99.29%	16.56%	99.92%	0.3193	62.50%

^a^Motif 1: X{4,13}SX[ST]; Motif 2: [VILMFWA][VILMFWA]XSX[ST][DE][DE][DE]; Motif 3: [VI]X[VI][VI]; Motif 4: [IV][IV]X[IVL]; Motif 5: KX{3,5}[VI][IL][IL]XXX[DEQN][DE][DE]; Motif 6: [IVL][DE][IVL][DE][IVL]; Motif 7: [PILVM][ILVM]X[ILVM][DSE>]{3}. Motif 8: [PILVM][ILVM]DLT; Motif 9: [DSE]{3}[ILVM]X[ILVMF]{2}. For the comparison, we fixed the *Sp* values of GPS-SUMO to compare the *Sn* scores.

To further evaluate the accuracy of GPS-SUMO, an additional testing set was used. This test set contained 71 positive sumoylation sites and 1376 negative sites from the most recently collected data set (Supplementary Table S3). Due to the data limitation, an additional data set for SUMO interaction prediction was not available. In Figure 1D, the ROC curve of the LOO validation was drawn and the AUC was calculated as 0.8629. Thus, our results suggested that the GPS-SUMO is still robust and accurate for the prediction of new data.

## USAGE

**Figure 2.**
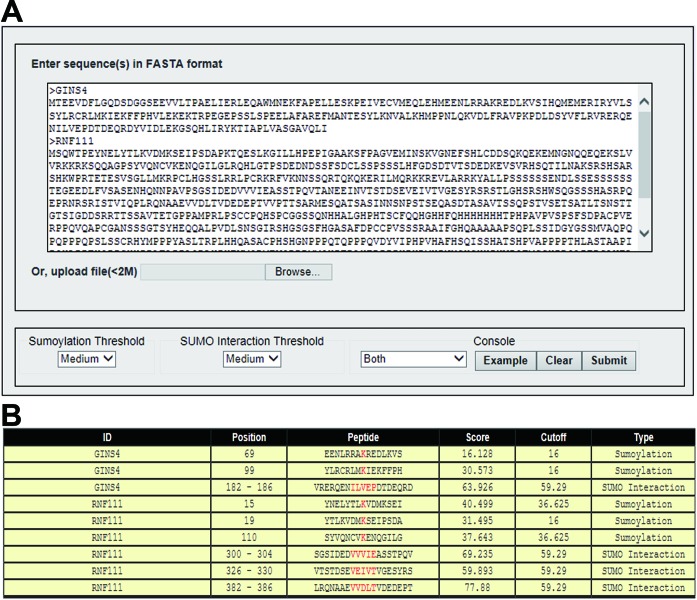
A snapshot of the GPS-SUMO web server. (**A**) As an example, the sequences of DNA replication complex GINS protein SLD5 (GINS4) and E3 ubiquitin-protein ligase Arkadia (RNF111) were inputted into GPS-SUMO. The sumoylation sites and SIMs were predicted using the medium threshold. (**B**) The prediction result of these two protein sequences. The information on the FASTA title, modified position, modified peptide, predicted score, prediction cutoff and regulation type are presented.

The online service of GPS-SUMO can predict both sumoylation sites and SIMs in a convenient manner (Figure 2A). In the console panel, a drop-down list was provided for selecting different prediction type. Also, an example button was provided. Two threshold panels for sumoylation and SUMO interaction prediction are located in the bottom left corner. Different prediction thresholds can be easily selected from the corresponding drop-down lists.

### Input description

In the web server page of GPS-SUMO, users can input one or multiple protein sequences with FASTA format in the text-box or upload a FASTA format file via the file selection dialog box. In order to guarantee a safe run of our web server, a maximal file size of 2M is allowed to be uploaded in each case. For large-scale predictions, a standalone program is available on the download page.

### Output description

All of the prediction results are presented in a tabular form containing the information of FASTA title, modified position, modified peptide, predicted score, prediction cutoff and modified type (Figure 2B). In the position column, the precise sumoylation sites as well as the SIMs are shown. Also, the predicted peptide for sumoylation or SUMO interaction is displayed in the peptide column with the sumoylation site or SIM shown in red. The cluster score and its corresponding cutoff are presented in the score and cutoff column, respectively. In the last column, the category of the SUMO regulation is indicated.

## DISCUSSION

GPS-SUMO is a novel web server that can be used to predict both covalent sumoylation sites and non-covalent SIMs. With a new generation GPS algorithm integrated with PSO method, the sumoylation site prediction performance was greatly improved. Also, by integrating a core hydrophobic motif of [IVL]{3,5}, GPS-SUMO achieves a precise prediction on SIMs. Taken together, we propose that GPS-SUMO will prove to be a highly useful web server in SUMO modification research. Moreover, the capability for predicting both sumoylation sites and SIMs in GPS-SUMO makes it potentially valuable for the investigation of the relationship between sumoylation and SUMO interaction.

During the training process, we classified all known sumoylation sites into two groups including the consensus group and non-consensus group based on the canonical motif of ψ–K–X–E. Although our algorithm generated satisfying performance on the consensus group, the performance for non-consensus group still remained to be improved. Therefore, in the subsequent release, a much more accurate GPS model will be applied in the prediction of the non-consensus sumoylation sites. As previously mentioned, the SIM has a distinct hydrophobic core. In this regard, a scoring strategy reflecting amino acid hydrophobicity will be further integrated to improve the accuracy of SIM prediction in the near future.

Previous studies revealed that serine and threonine residues are abundant in proximity to the hydrophobic core of a subset of SIMs, and raised a concern that whether phosphorylation plays an important role in the regulation of SUMO-interacting activity (15,35). Indeed, the E3 ligase PIASxα is phosphorylated *in vivo* at serines adjacent to the hydrophobic core of the SIM, and the PIASxα phosphorylation modulates its interacting to SUMO1 but not to SUMO2 (15). By searching several public databases of the phosphorylation, including Phospho.ELM (36), PhosphoSitePlus (37) and PhosphoGRID (38), we totally obtained 1016 non-redundant phosphorylation sites in 80 known SUMO-interacting proteins. Then we looked through the flanking regions such as (5, 5), (10, 10) and (15, 15) with at least one phosphorylation site around known SIMs and negative peptides following the [IVL]{3,5} motif (Table 2). Using the Chi-Squared Test (Right-Tail) (39), we observed that phosphorylation sites are significantly over-represented in adjacent regions of known SIMs against negative peptides (Table 2, *P*-value << 0.01). Totally, there were 20 (14.6%) and 27 (19.7%) known SIMs with at least one phosphorylation sites in the adjacent regions of (5, 5) and (10, 10), respectively (Table 2). When the flanking region was extended to (15, 15), the number of SIMs with flanking phosphorylation sites was only moderately increased, while the level of significance was decreased. In this regard, although the phosphorylation information was not integrated into GPS-SUMO, at least a known phosphorylation site in the (10, 10) flanking region of the SIM can be a good indicator for further identifying phospho-regulated SIMs.

**Table 2.** Known phosphorylation sites in adjacent regions of known SIMs and negative peptides following the [IVL]{3,5} motif

Flanking	Known SIMs		[IVL]{3,5} negative^a^		
	Phos.^b^	Other^c^	Phos.	Other	*P*-value^d^
(5,5)	20	117	50	1649	7.23E-12
(10,10)	27	110	93	1606	8.90E-11
(15,15)	30	107	134	1565	3.18E-08

^a^The negative data set with peptide following the [IVL]{3,5} motif.

^b^The number of SIMs with at least one phosphorylation site in flanking regions.

^c^The number of SIMs without any adjacent phosphorylation sites.

^d^The *P*-value was calculated with the Chi-squared test.

## SUPPLEMENTARY DATA

Supplementary Data are available at NAR Online, including [1–6].

Supplementary Data

## References

[1] Geiss-Friedlander R., Melchior F. (2007). Concepts in sumoylation: a decade on. Nat. Rev. Mol. Cell Biol..

[2] Hay R.T. (2005). SUMO: a history of modification. Mol. Cell.

[3] Muller S., Hoege C., Pyrowolakis G., Jentsch S. (2001). SUMO, ubiquitin's mysterious cousin. Nat. Rev. Mol. Cell Biol..

[4] Seeler J.S., Dejean A. (2003). Nuclear and unclear functions of SUMO. Nat. Rev. Mol. Cell Biol..

[5] Lee L., Sakurai M., Matsuzaki S., Arancio O., Fraser P. (2013). SUMO and Alzheimer's disease. Neuromolecular Med..

[6] Eckermann K. (2013). SUMO and Parkinson's disease. Neuromolecular Med..

[7] Wang J., Chen L., Wen S., Zhu H., Yu W., Moskowitz I.P., Shaw G.M., Finnell R.H., Schwartz R.J. (2011). Defective sumoylation pathway directs congenital heart disease. Birth Defects Res. A Clin. Mol. Teratol..

[8] Zhao J. (2007). Sumoylation regulates diverse biological processes. Cell. Mol. Life Sci..

[9] Seeler J.S., Bischof O., Nacerddine K., Dejean A. (2007). SUMO, the three Rs and cancer. Curr. Top. Microbiol. Immunol..

[10] Gill G. (2005). Something about SUMO inhibits transcription. Curr. Opin. Genet. Dev..

[11] Melchior F. (2000). SUMO–nonclassical ubiquitin. Annu. Rev. Cell Dev. Biol..

[12] Rodriguez M.S., Dargemont C., Hay R.T. (2001). SUMO-1 conjugation in vivo requires both a consensus modification motif and nuclear targeting. J. Biol. Chem..

[13] Sampson D.A., Wang M., Matunis M.J. (2001). The small ubiquitin-like modifier-1 (SUMO-1) consensus sequence mediates Ubc9 binding and is essential for SUMO-1 modification. J. Biol. Chem..

[14] Hannich J.T., Lewis A., Kroetz M.B., Li S.J., Heide H., Emili A., Hochstrasser M. (2005). Defining the SUMO-modified proteome by multiple approaches in Saccharomyces cerevisiae. J. Biol. Chem..

[15] Hecker C.M., Rabiller M., Haglund K., Bayer P., Dikic I. (2006). Specification of SUMO1- and SUMO2-interacting motifs. J. Biol. Chem..

[16] Kerscher O., Felberbaum R., Hochstrasser M. (2006). Modification of proteins by ubiquitin and ubiquitin-like proteins. Annu. Rev. Cell Dev. Biol..

[17] Lin D.Y., Huang Y.S., Jeng J.C., Kuo H.Y., Chang C.C., Chao T.T., Ho C.C., Chen Y.C., Lin T.P., Fang H.I. (2006). Role of SUMO-interacting motif in Daxx SUMO modification, subnuclear localization, and repression of sumoylated transcription factors. Mol. Cell.

[18] Ouyang J., Shi Y., Valin A., Xuan Y., Gill G. (2009). Direct binding of CoREST1 to SUMO-2/3 contributes to gene-specific repression by the LSD1/CoREST1/HDAC complex. Mol. Cell.

[19] Minty A., Dumont X., Kaghad M., Caput D. (2000). Covalent modification of p73alpha by SUMO-1. Two-hybrid screening with p73 identifies novel SUMO-1-interacting proteins and a SUMO-1 interaction motif. J. Biol. Chem..

[20] Song J., Durrin L.K., Wilkinson T.A., Krontiris T.G., Chen Y. (2004). Identification of a SUMO-binding motif that recognizes SUMO-modified proteins. Proc. Natl. Acad. Sci. U.S.A..

[21] Song J., Zhang Z., Hu W., Chen Y. (2005). Small ubiquitin-like modifier (SUMO) recognition of a SUMO binding motif: a reversal of the bound orientation. J. Biol. Chem..

[22] Vogt B., Hofmann K. (2012). Ubiquitin Family Modifiers and the Proteasome.

[23] Husnjak K., Dikic I. (2012). Ubiquitin-binding proteins: decoders of ubiquitin-mediated cellular functions. Annu. Rev. Biochem..

[24] Dikic I., Wakatsuki S., Walters K.J. (2009). Ubiquitin-binding domains — from structures to functions. Nat. Rev. Mol. Cell Biol..

[25] Xue Y., Zhou F., Fu C., Xu Y., Yao X. (2006). SUMOsp: a web server for sumoylation site prediction. Nucleic Acids Res..

[26] Ren J., Gao X., Jin C., Zhu M., Wang X., Shaw A., Wen L., Yao X., Xue Y. (2009). Systematic study of protein sumoylation: development of a site-specific predictor of SUMOsp 2.0. Proteomics.

[27] Teng S., Luo H., Wang L. (2012). Predicting protein sumoylation sites from sequence features. Amino Acids.

[28] Xu J., He Y., Qiang B., Yuan J., Peng X., Pan X.M. (2008). A novel method for high accuracy sumoylation site prediction from protein sequences. BMC Bioinformatics.

[29] Chen Y.Z., Chen Z., Gong Y.A., Ying G. (2012). SUMOhydro: a novel method for the prediction of sumoylation sites based on hydrophobic properties. PLoS One.

[30] Eberhart R.C., Kennedy J. (1995). A new optimizer using particle swarm theory. Proceedings of the Sixth International Symposium on Micro Machine and Human Science.

[31] Kennedy J., Eberhart R.C. (1997). A discrete binary version of the particle swarm algorithm. Systems, Man, and Cybernetics, 1997. 1997 IEEE International Conference on Computational Cybernetics and Simulation..

[32] Li W., Godzik A. (2006). Cd-hit: a fast program for clustering and comparing large sets of protein or nucleotide sequences. Bioinformatics.

[33] Xue Y., Liu Z., Gao X., Jin C., Wen L., Yao X., Ren J. (2010). GPS-SNO: computational prediction of protein S-nitrosylation sites with a modified GPS algorithm. PloS One.

[34] Crooks G.E., Hon G., Chandonia J.M., Brenner S.E. (2004). WebLogo: a sequence logo generator. Genome Res..

[35] Zhu J., Zhu S., Guzzo C.M., Ellis N.A., Sung K.S., Choi C.Y., Matunis M.J. (2008). Small ubiquitin-related modifier (SUMO) binding determines substrate recognition and paralog-selective SUMO modification. J. Biol. Chem..

[36] Dinkel H., Chica C., Via A., Gould C.M., Jensen L.J., Gibson T.J., Diella F. (2011). Phospho.ELM: a database of phosphorylation sites–update 2011. Nucleic Acids Res..

[37] Hornbeck P.V., Kornhauser J.M., Tkachev S., Zhang B., Skrzypek E., Murray B., Latham V., Sullivan M. (2012). PhosphoSitePlus: a comprehensive resource for investigating the structure and function of experimentally determined post-translational modifications in man and mouse. Nucleic Acids Res..

[38] Stark C., Su T.C., Breitkreutz A., Lourenco P., Dahabieh M., Breitkreutz B.J., Tyers M., Sadowski I. (2010). PhosphoGRID: a database of experimentally verified in vivo protein phosphorylation sites from the budding yeast Saccharomyces cerevisiae. Database.

[39] Greenwood P.E., Nikulin M.S. (1996). A Guide to Chi-squared Testing.

