# Supplementary material for: GPS-SUMO: a tool for the prediction of sumoylation sites and SUMO-interaction motifs
Source: Nucleic Acids Res. 2014 May 31;42(Web Server issue):W325–30. doi: 10.1093/nar/gku383 (PMC4086084; doi:10.1093/nar/gku383)
Supplement: Supplementary Data [file supp_42_W1_W325__index.html]

Supplementary Data 

# GPS-SUMO: a tool for the prediction of sumoylation sites and SUMO-interaction motifs

## Supplementary Data

**Files in this Data Supplement:**

- SUPPLEMENTARY DATA
