# Supplementary material for: GPS-SUMO: a tool for the prediction of sumoylation sites and SUMO-interaction motifs
Source: Nucleic Acids Res. 2014 May 31;42(Web Server issue):W325–30. doi: 10.1093/nar/gku383 (PMC4086084; doi:10.1093/nar/gku383)
Supplement: Supplementary Data [file supp_gku383_nar-00459-web-b-2014-File004.zip › Supplementary Data.pdf]

Supplementary Data

GPS-SUMO: a tool for the prediction of sumoylation sites and SUMO-interaction motifs

Qi Zhao[1,†], Yubin Xie[1,†], Yueyuan Zheng[1,†], Shuai Jiang[1], Wenzhong Liu[1], Weiping Mu[1], Zexian Liu[2], Yong Zhao[1], Yu Xue[2,*] and Jian Ren[1,*]

[1]State Key Laboratory of Biocontrol, School of Life Sciences, School of Advanced Computing, Sun Yat-set University, Guangzhou 510275, China

[2]Department of Biomedical Engineering, College of Life Science and Technology, Huazhong University of Science and Technology, Wuhan 430074, China

Running title: The GPS-SUMO web server

*To whom correspondence should be addressed. Tel/Fax:+86 20 39943788; Email: renjian.sysu@gmail.com;

Correspondence may also be addressed to Yu Xue. Tel:+86 27 87793903; Fax:+86 27 87793172; Email: xueyu@hust.edu.cn.

[†]The authors wish it to be known that, in their opinion, the first three authors should be regarded as joint First Authors.

Supplementary Methods

The GPS algorithm

The fourth-generation Group-based Prediction System (GPS) algorithm was employed in GPS-SUMO. Based on the hypothesis that short peptides with high sequence homology would have similar biochemical properties (1), a group-based scoring strategy was applied to calculate the similarity score between two sumoylation or SUMO interaction peptides. As previously described (2), a potential sumoylation peptide *PSP(m, n)* is defined as a sumoylated lysine residue flanked by *m* residues upstream and *n* residues downstream. For the prediction of SUMO-interaction motifs (SIMs), we summarized a hydrophobic motif of [IVL]{3,5} from previously identified SIMs. According to the summarized motif, a pentapeptide with at least three hydrophobic residues (*e.g.* I, V, and L) is regarded as a potential SIM. Based on this definition, a potential SIM peptide *PSIP(m, n)* can be obtained as a SIM flanked by *m* residues upstream and *n* residues downstream. Once the *PSP(m, n)* and *PSIP(m, n)* are defined, the similarity score of two *PSP(m, n)* items or two *PSIP(m, n)* items may be calculated as shown in Eq. 1:

$$S(A, B) = \sum_{-m \le i \le n} Score(A_i, B_i) \qquad \text{Eq. 1}$$

where *Score* (A_i, B_i) represents the substitution score for the two amino acids A_i and B_i in the BLOSUM62 matrix. *S(A,B)* is redefined as zero when $S(A, B) \le 0$. In fact, one type of post-translational modification (PTM) is capable of recognizing multiple motifs. Therefore, the *PSP(m, n)* or *PSIP(m, n)* items from the training data set were classified into several different groups based on recognition motifs. In this work, the *PSP(m, n)* items were classified into a consensus group and non-consensus group based on the ψ-K-X-E motif. Due to data limitations, the *PSIP(m, n)* samples were simply clustered into one group. When a *PSP(m, n)* or *PSIP(m, n)* is given, the GPS algorithm calculates the average score between the peptide and the experimentally verified sumoylation or SUMO interaction peptides in each cluster. If the average score is larger than a preset threshold, the corresponding site is predicted as a sumoylation site or SIM. To improve the sensitivity of the GPS algorithm, a simple approach of filtering out noise was implemented. When calculating the average score, experimentally verified peptides with a similarity score lower than zero were discarded so that the discrete peptides (*i.e.*, “noise”) were effectively eliminated. Alternately, in consideration of the risk in overestimation, a

minimal size was assigned so as to put a limit on the smallest number of peptides in one cluster. Furthermore, the scoring strategy can be improved by three sequential training steps: peptide selection, weight training and matrix mutation.

(1) *k*-means clustering. In this work, the *k*-means clustering was adopted to classify the non-consensus sumoylation sites. When two *PSP(m, n)* is given, the similarity score can be calculated using Eq. 2:

$$S(A, B) = \frac{Num.of\ conserved\ substitutions}{Num.of\ all\ substitutions} \qquad \text{Eq.2}$$

A conserved substitution is a substitution with a *Score(a, b)>0* in the BLOSUM62 matrix. The *S(A, B)* ranges from 0 to 1. The distance between the two *PSP(m, n)* is then defined as: *D(A,B)=1/S(A,B)*. If *S(A,B)=0*, we simply let *D(A,B)=∞*. The *k*-means algorithm clusters the non-consensus sumoylation sites by exhaustive testing. First of all, two sumoylation sites were randomly chosen as the centroids. Secondly, other positive sites were compared with the two centroids and the distances were calculated. With the shortest distance, the positive sites were then clustered into the corresponding groups. Thirdly, the centroids were updated with the highest average identity score. Optimal cluster can be obtained by iterative repeat of the second and third steps. After the clusters for positive sites have been determined, the negative sites following the ψ-K-X-E motif were regarded as the negative sites for the consensus group, while remaining negative sites were put into the other clusters based on the highest average similarity scores. As a result, the non-consensus sumoylation sites were clustered into two distinct groups.

(2) Motif Length Selection (MLS). The peptide selection step singles out the optimal combination of *PSP(m, n)* and *PSIP(m, n)* for each peptide group. In an extensive test, the leave-one-out (LOO) validations for all of the combinations of *PSP(m, n)* and *PSIP(m, n)* (*m*=1…30, *n*=1…30) were carried out. In this study, the Specificity value (*Sp*) was fixed at 90% for sumoylation and 95% for SUMO interaction to select the optimal *PSP(m, n)* and *PSIP(m, n)* with the highest Sensitivity (*Sn*) value.

(3) Weight Training (WT). To evaluate the amino acid preference of the modified enzymes, a weight training method was adopted to optimize the scoring weight. In weight training, the PSO method (3,4) was integrated to search for a set of optimal scoring weights that maximize the *Sn* value in terms of LOO validation. After the weight training process, the scoring strategy was redefined as shown in Eq. 3:

$$S(A, B) = \sum_{-m \le i \le n} w_i Score(A_i, B_i) \qquad \text{Eq. 3}$$

where *wi* refers to the scoring weight of each position.

(4) Matrix Mutation (MaM). It was previously shown that the Matrix Mutation approach efficiently enhances the performance of GPS prediction (1). Therefore, the Matrix Mutation approach was adopted in this work. Similar to weight training, we used the PSO algorithm to select an optimal substitute substitution matrix for SUMO modification. The LOO validation was used as a fitness function in the PSO. During the training step, the *Sn* value was fixed at 90% for sumoylation and 95% for SUMO interaction.

(5) Particle swarm optimization (PSO). In the previous version (2), the WT and MaM steps were implemented in a random mutation algorithm that required repeated training and resulted in a low convergence rate. Thus, in the fourth-generation GPS algorithm, the PSO method (3,4) was integrated. Before the optimization steps, we re-illustrated the weight training process as shown in Eq. 4:

$$w_i = 1 + \Delta w_i \qquad \text{Eq. 4}$$

where w_i is the scoring weight, and Δw_i represents the numeric changes in the scoring weight after the training process. Therefore, the WT process is aimed at finding the set of Δw_i that obtains the best performance. Similarly, the MaM process can also be described, as shown in Eq. 5:

$$S(a, b) = Score(a, b) + \Delta S(a, b) \qquad \text{Eq. 5}$$

where *S(a, b)* is the optimal substitution score for the amino acids *a* and *b* with respect to SUMO modification. *Score(a, b)* is the substitution score in the BLOSUM62 matrix. *ΔS(a, b)* represents the numeric changes in substitution score for amino acids *a* and *b*. Thus, the MaM approach seeks for a set of *ΔS(a, b)* that maximizes the prediction performance.

The first step of PSO begins by transforming the candidate solution of the problem into a set of particles. For each particle, it is comprised of three D-dimensional vectors, where D is the search space. These three vectors are the current position x_i, the previous best position p_i and the velocity v_i. The PSO then initializes a population array of particles with random current positions and velocities on D-dimensions. In this case, the randomly generated Δw_i and *ΔS(a, b)* are directly assigned to x_i. For each particle, the leave-one-out (LOO) validation is used to evaluate the prediction performance in the current position. Next, the current position is compared to the previous best position. If the current position is superior to the previous best position, the p_i is set equal to the current location x_i. To

simulate the process of information exchange, each particle identifies another particle in its neighborhood that has the current best position. In this implementation, the neighborhood structure of each particle is defined as a ring topology (5). The previous best position p_i for that best neighbor is then stored in the best neighbor position p_g. After finding the p_g, the velocity and the current position are adjusted according to Eq. 6:

$$\begin{cases} v_i = \omega \times v_i + Rand(0, \varphi_1) \otimes (p_i - x_i) + Rand(0, \varphi_2) \otimes (p_g - x_i) \\ x_i = x_i + v_i \end{cases} \qquad \text{Eq. 6}$$

where ω represents as inertia weight, and $Rand(0,\varphi_1)$ and $Rand(0,\varphi_2)$ are two random functions generated real numbers distributed in $[0,\varphi_1]$ and $[0,\varphi_2]$. $\otimes$ is the component-wise multiplication. Notably, the range of each v_i is limited between $-V_{max}$ and $+V_{max}$. Based on the best position found in a specific neighborhood, a particle moves to a new position that much closer to the globally optimal one. With this approach, each particle communicates with other particles and collaboratively seeks the optimal solution. To obtain the optimal solution, the PSO iteratively performs the steps described above until a convergence criterion is met.

Supplementary References

1. Xue, Y., Ren, J., Gao, X., Jin, C., Wen, L. and Yao, X. (2008) GPS 2.0, a tool to predict kinase-specific phosphorylation sites in hierarchy. *Molecular & Cellular Proteomics*, **7**, 1598-1608.
2. Xue, Y., Liu, Z., Gao, X., Jin, C., Wen, L., Yao, X. and Ren, J. (2010) GPS-SNO: computational prediction of protein S-nitrosylation sites with a modified GPS algorithm. *PloS one*, **5**, e11290.
3. Eberhart, R.C. and Kennedy, J. (1995) A new optimizer using particle swarm theory. *Proceedings of the sixth international symposium on micro machine and human science*, **1**, 39-43.
4. Kennedy, J. and Eberhart, R.C. (1997) A discrete binary version of the particle swarm algorithm. *Systems, Man, and Cybernetics, 1997. Computational Cybernetics and Simulation., 1997 IEEE International Conference on*, **5**, 4104-4108.
5. Ochoa, A., Hernández, A., Cruz, L., Ponce, J., Montes, F., Li, L. and Janacek, L. (2010) Artificial Societies and Social Simulation using Ant Colony, Particle Swarm Optimization and Cultural Algorithms.
6. Crooks, G.E., Hon, G., Chandonia, J.M. and Brenner, S.E. (2004) WebLogo: a sequence logo generator. *Genome research*, **14**, 1188-1190.

Supplementary Figures

Supplementary Figure S1 – The sequence logos for three clusters of sumoylation sites. The PSP(15, 15) items of each cluster were submitted into Weblogo 2.8.2 (6), separately. (A) The consensus sites follow the ψ-K-X-E motif. (B) The sites following the ψ-K-X-D motif are highly enriched. (C) The sequence profile is elusive.

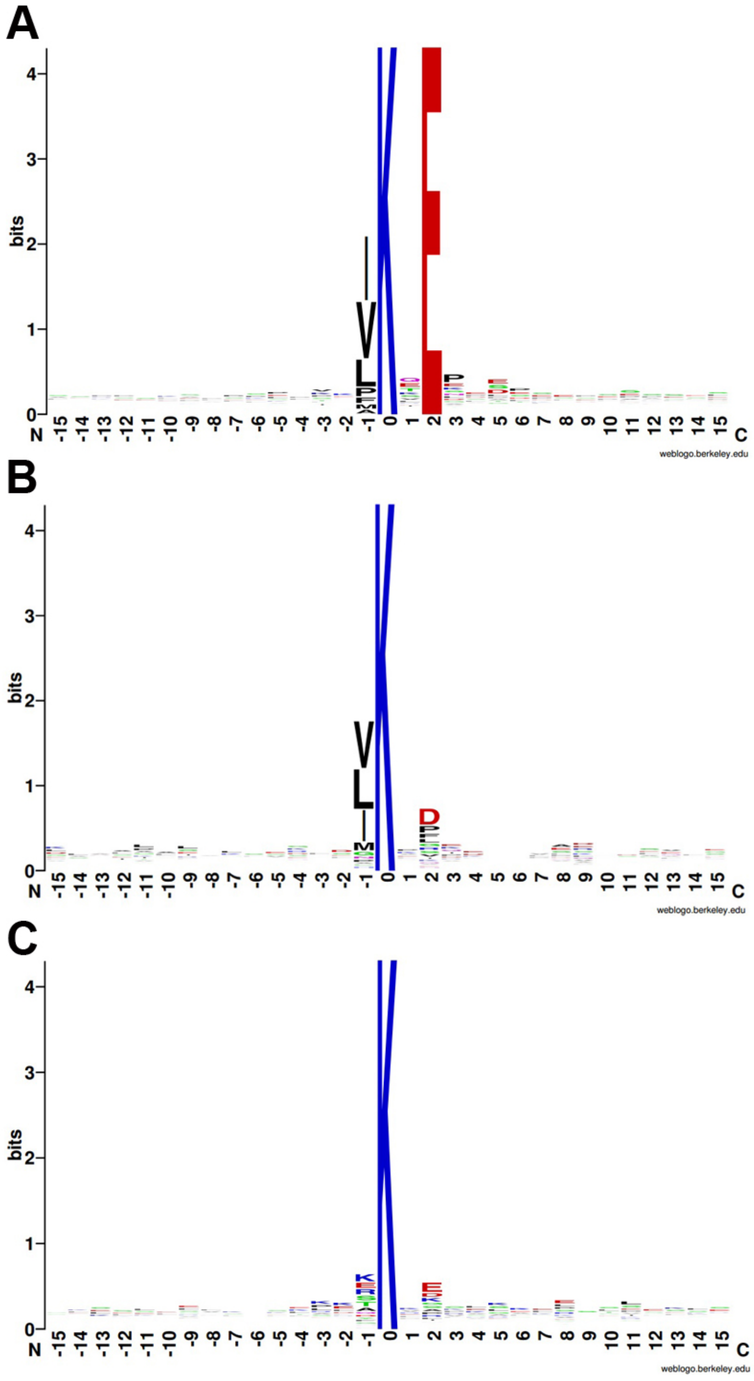

Supplementary Tables

Supplementary Table S1 – The sumoylation training data set. This table lists the non-redundant data set used in sumoylation training. The UniProt accessions, precise modified sites and sumoylation peptides are included.

Supplementary Table S2 – The SUMO interaction training data set. Similar to Table S1, the table lists the non-redundant data set used in SUMO interaction training.

Supplementary Table S3 – An additional test set. The additional test set used in the sumoylation evaluation is included in this table.
